# 6-(2,6-Dimethyl­phen­yl)pyrido[2,3-*d*]pyrimidin-7-amine

**DOI:** 10.1107/S1600536809006242

**Published:** 2009-02-25

**Authors:** Seiji Nukui, Arnold L. Rheingold, Antonio DiPasquale, Alex Yanovsky

**Affiliations:** a Pfizer Global Research and Development, La Jolla Labs, 10614 Science Center Drive, San Diego, CA 92121, USA; b Department of Chemistry and Biochemistry, University of California, San Diego, 9500 Gilman Drive, La Jolla, CA 92093, USA

## Abstract

In the title compound, C_15_H_14_N_4_, the pyrido[2,3-*d*]pyrimidine system is almost ideally planar (r.m.s. deviation 0.028 Å) with its mean plane almost orthogonal to the 2,6-dimethyl­phenyl plane. The dihedral angle formed by these planes [87.3 (2)°] is close to the predicted value (89.7°) obtained by mol­ecular-mechanics force-field calculations. Only one of the two active amine H atoms participates in hydrogen bonding, which links mol­ecules into centrosymmetric dimers.

## Related literature

For the structures of related pyrido[2,3-*d*]pyrimidine derivatives, see: Hamby *et al.* (1997 ▶); Trumpp-Kallmeyer *et al.* (1998 ▶). For the synthesis of the title compound, see: Bennett *et al.* (1981 ▶); Blankley & Bennett (1981 ▶). For mol­ecular-mechanics force-field calculations, see: Duan *et al.* (2003 ▶).
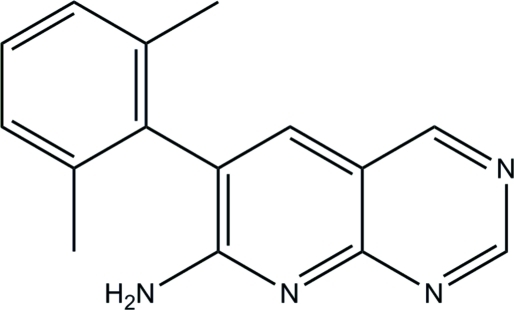


## Experimental

### 

#### Crystal data

C_15_H_14_N_4_
*M* *_r_* = 250.30
Monoclinic, 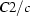

*a* = 16.272 (3) Å
*b* = 10.644 (2) Å
*c* = 15.234 (3) Å
β = 109.118 (3)°
*V* = 2493.0 (8) Å^3^
*Z* = 8
Mo *K*α radiation
μ = 0.08 mm^−1^
*T* = 208 K
0.14 × 0.06 × 0.06 mm

#### Data collection

Bruker Kappa APEXII CCD area-detector diffractometer
Absorption correction: multi-scan (*SADABS*; Bruker, 2001 ▶) *T*_min_ = 0.988, *T*_max_ = 0.995
6161 measured reflections
2863 independent reflections
1706 reflections with *I* > 2σ(*I*)
*R*_int_ = 0.037

#### Refinement

*R*[*F* ^2^ > 2σ(*F* ^2^)] = 0.054
*wR*(*F* ^2^) = 0.152
*S* = 0.98
2863 reflections
174 parameters
H-atom parameters constrained
Δρ_max_ = 0.22 e Å^−3^
Δρ_min_ = −0.22 e Å^−3^

### 

Data collection: *APEX2* (Bruker, 2004 ▶); cell refinement: *SAINT* (Bruker, 2004 ▶); data reduction: *SAINT*; program(s) used to solve structure: *SIR2004* (Burla *et al.*, 2005 ▶); program(s) used to refine structure: *SHELXL97* (Sheldrick, 2008 ▶); molecular graphics: *ORTEP-32* (Farrugia, 1997 ▶); software used to prepare material for publication: *WinGX* (Farrugia, 1999 ▶).

## Supplementary Material

Crystal structure: contains datablocks global, I. DOI: 10.1107/S1600536809006242/rz2295sup1.cif

Structure factors: contains datablocks I. DOI: 10.1107/S1600536809006242/rz2295Isup2.hkl

Additional supplementary materials:  crystallographic information; 3D view; checkCIF report

## Figures and Tables

**Table 1:** Hydrogen-bond geometry (Å, °)

*D*—H⋯*A*	*D*—H	H⋯*A*	*D*⋯*A*	*D*—H⋯*A*
N4—H4*A*⋯N1^i^	0.87	2.18	3.044 (2)	171

Symmetry code: (i) 
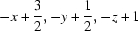
.

**Fig. 1.**
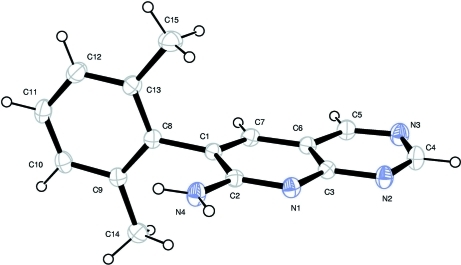
Molecular structure of the title compound, showing 50% probability displacement ellipsoids and atom numbering scheme. H atoms are drawn as circles with arbitrary small radius.

**Fig. 2.**
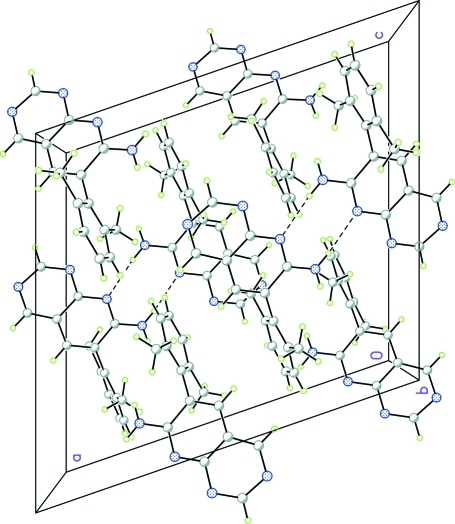
The crystal packing diagram viewed down the b-axis; H-bonds are shown as dashed lines.

## supplementary crystallographic information

### Comment

The present X-ray study confirmed the structure of the compound reported in Bennett *et al.* (1981) as 6-(2,6-dimethylphenyl)pyrido[2,3-*d*]pyrimidin-7-amine. The pyrido[2,3-*d*]pyrimidine system of the molecule of the title compound (Fig. 1) is planar within 0.045 Å. The 2,6-dimethylphenyl plane is approximately orthogonal to the mean plane of the bicyclic system; the corresponding dihedral angle [87.3 (2)°] is close to predicted value (89.7°), obtained by molecular mechanics force field calculations (Duan *et al.*, 2003). The overall geometry of the molecule is quite close to the structures of previously studied phenyl substituted 7-aminopyrido[2,3-*d*]pyrimidines (Hamby *et al.*, 1997; Trumpp-Kallmeyer *et al.*, 1998).

Only one of the two amine H atoms (H4A) participates in the H-bonding (Table 1), which is responsible for formation of centrosymmetric dimers in the crystal. The H4B atom is close to the π-electron density of the phenyl ring and is not involved in either intra- or intermolecular H-bonding.

### Experimental

The title compound was synthesized according to Bennett *et al.* (1981) and Blankley & Bennett (1981).

### Refinement

All H atoms were treated as riding with the C—H(aromatic), *C*—*H*(methyl) and N—H distances of 0.94 Å, 0.97 Å and 0.87 Å respectively; the *U*_iso_(H) were set to 1.2*U*_eq_ of the carrying atom for aromatic and amine, and 1.5*U*_eq_ for methyl H atoms.

### Crystal data

**Table:**

C_15_H_14_N_4_	*F*(000) = 1056
*M**_r_* = 250.30	*D*_x_ = 1.334 Mg m^−^^3^
Monoclinic, *C*2/*c*	Mo *K*α radiation, λ = 0.71073 Å
Hall symbol: -C 2yc	Cell parameters from 1859 reflections
*a* = 16.272 (3) Å	θ = 2.3–27.8°
*b* = 10.644 (2) Å	µ = 0.08 mm^−^^1^
*c* = 15.234 (3) Å	*T* = 208 K
β = 109.118 (3)°	Block, colorless
*V* = 2493.0 (8) Å^3^	0.14 × 0.06 × 0.06 mm
*Z* = 8	

### Data collection

**Table:**

Bruker Kappa-APEX2 CCD area-detector diffractometer	2863 independent reflections
Radiation source: fine-focus sealed tube	1706 reflections with *I* > 2σ(*I*)
graphite	*R*_int_ = 0.037
φ and ω scans	θ_max_ = 28.2°, θ_min_ = 2.3°
Absorption correction: multi-scan (*SADABS*; Bruker, 2001)	*h* = −21→21
*T*_min_ = 0.988, *T*_max_ = 0.995	*k* = −13→10
6161 measured reflections	*l* = −11→20

### Refinement

**Table:**

Refinement on *F*^2^	Primary atom site location: structure-invariant direct methods
Least-squares matrix: full	Secondary atom site location: difference Fourier map
*R*[*F*^2^ > 2σ(*F*^2^)] = 0.054	Hydrogen site location: inferred from neighbouring sites
*wR*(*F*^2^) = 0.152	H-atom parameters constrained
*S* = 0.98	*w* = 1/[σ^2^(*F*_o_^2^) + (0.0773*P*)^2^] where *P* = (*F*_o_^2^ + 2*F*_c_^2^)/3
2863 reflections	(Δ/σ)_max_ < 0.001
174 parameters	Δρ_max_ = 0.22 e Å^−^^3^
0 restraints	Δρ_min_ = −0.22 e Å^−^^3^

### Special details

**Table:**

Geometry. All e.s.d.'s (except the e.s.d. in the dihedral angle between two l.s. planes) are estimated using the full covariance matrix. The cell e.s.d.'s are taken into account individually in the estimation of e.s.d.'s in distances, angles and torsion angles; correlations between e.s.d.'s in cell parameters are only used when they are defined by crystal symmetry. An approximate (isotropic) treatment of cell e.s.d.'s is used for estimating e.s.d.'s involving l.s. planes.
Refinement. Refinement of *F*^2^ against ALL reflections. The weighted *R*-factor *wR* and goodness of fit *S* are based on *F*^2^, conventional *R*-factors *R* are based on *F*, with *F* set to zero for negative *F*^2^. The threshold expression of *F*^2^ > σ(*F*^2^) is used only for calculating *R*-factors(gt) *etc*. and is not relevant to the choice of reflections for refinement. *R*-factors based on *F*^2^ are statistically about twice as large as those based on *F*, and *R*- factors based on ALL data will be even larger.

### Fractional atomic coordinates and isotropic or equivalent isotropic displacement parameters (Å^2^)

**Table:**

	*x*	*y*	*z*	*U*_iso_*/*U*_eq_	
C1	0.60306 (12)	0.28432 (17)	0.63650 (12)	0.0292 (4)	
C2	0.66357 (12)	0.27235 (16)	0.58459 (13)	0.0286 (4)	
C3	0.56152 (12)	0.17232 (17)	0.46410 (13)	0.0298 (4)	
C4	0.46019 (14)	0.0833 (2)	0.34015 (15)	0.0425 (5)	
H4	0.4461	0.0508	0.2796	0.051*	
C5	0.41654 (13)	0.12949 (19)	0.46163 (14)	0.0386 (5)	
H5	0.3743	0.1300	0.4914	0.046*	
C6	0.49866 (12)	0.17682 (17)	0.50973 (13)	0.0309 (4)	
C7	0.52301 (12)	0.23505 (18)	0.59840 (13)	0.0323 (5)	
H7	0.4829	0.2392	0.6308	0.039*	
C8	0.62801 (12)	0.35600 (18)	0.72563 (13)	0.0302 (4)	
C9	0.66754 (12)	0.29645 (19)	0.81040 (13)	0.0322 (5)	
C10	0.68565 (13)	0.3677 (2)	0.89141 (14)	0.0389 (5)	
H10	0.7113	0.3285	0.9493	0.047*	
C11	0.66687 (14)	0.4936 (2)	0.88847 (15)	0.0456 (6)	
H11	0.6798	0.5399	0.9439	0.055*	
C12	0.62906 (14)	0.5519 (2)	0.80438 (15)	0.0451 (6)	
H12	0.6164	0.6382	0.8028	0.054*	
C13	0.60939 (12)	0.48549 (19)	0.72209 (13)	0.0355 (5)	
C14	0.69087 (15)	0.1587 (2)	0.81607 (15)	0.0472 (6)	
H14A	0.6435	0.1098	0.8241	0.071*	
H14B	0.7433	0.1446	0.8685	0.071*	
H14C	0.7008	0.1331	0.7592	0.071*	
C15	0.56830 (15)	0.5511 (2)	0.63050 (15)	0.0500 (6)	
H15A	0.5664	0.6408	0.6409	0.075*	
H15B	0.5097	0.5197	0.6016	0.075*	
H15C	0.6024	0.5350	0.5901	0.075*	
N1	0.64297 (10)	0.21747 (15)	0.50164 (10)	0.0315 (4)	
N2	0.54103 (11)	0.12193 (16)	0.37767 (11)	0.0377 (4)	
N3	0.39463 (11)	0.08379 (17)	0.37626 (12)	0.0435 (5)	
N4	0.74304 (10)	0.32180 (15)	0.61956 (11)	0.0375 (4)	
H4A	0.7791	0.3174	0.5883	0.045*	
H4B	0.7587	0.3584	0.6736	0.045*	

### Atomic displacement parameters (Å^2^)

**Table:**

	*U*^11^	*U*^22^	*U*^33^	*U*^12^	*U*^13^	*U*^23^
C1	0.0304 (10)	0.0319 (10)	0.0273 (10)	0.0037 (8)	0.0122 (8)	0.0018 (8)
C2	0.0303 (10)	0.0287 (10)	0.0283 (10)	0.0014 (8)	0.0115 (8)	0.0037 (8)
C3	0.0311 (10)	0.0302 (10)	0.0292 (10)	−0.0008 (8)	0.0116 (8)	0.0015 (8)
C4	0.0454 (13)	0.0515 (14)	0.0317 (11)	−0.0075 (10)	0.0141 (10)	−0.0073 (10)
C5	0.0325 (11)	0.0459 (12)	0.0393 (12)	−0.0015 (9)	0.0142 (9)	−0.0036 (10)
C6	0.0317 (10)	0.0326 (11)	0.0305 (10)	0.0015 (8)	0.0129 (8)	0.0018 (9)
C7	0.0303 (11)	0.0379 (11)	0.0340 (11)	0.0029 (8)	0.0178 (9)	0.0010 (9)
C8	0.0259 (10)	0.0378 (11)	0.0312 (10)	−0.0020 (8)	0.0151 (8)	−0.0034 (9)
C9	0.0281 (10)	0.0373 (11)	0.0330 (11)	−0.0026 (8)	0.0124 (9)	−0.0010 (9)
C10	0.0337 (11)	0.0530 (14)	0.0303 (11)	−0.0046 (9)	0.0107 (9)	−0.0028 (10)
C11	0.0475 (13)	0.0527 (15)	0.0372 (13)	−0.0062 (10)	0.0144 (10)	−0.0156 (11)
C12	0.0474 (13)	0.0382 (12)	0.0508 (14)	0.0008 (10)	0.0176 (11)	−0.0090 (11)
C13	0.0358 (11)	0.0356 (11)	0.0374 (12)	−0.0009 (9)	0.0149 (9)	−0.0018 (9)
C14	0.0541 (14)	0.0454 (14)	0.0386 (12)	0.0074 (10)	0.0105 (10)	0.0038 (10)
C15	0.0591 (15)	0.0417 (13)	0.0499 (14)	0.0078 (11)	0.0188 (12)	0.0070 (11)
N1	0.0316 (9)	0.0381 (9)	0.0289 (9)	−0.0014 (7)	0.0156 (7)	−0.0005 (7)
N2	0.0390 (10)	0.0462 (10)	0.0306 (9)	−0.0056 (8)	0.0150 (8)	−0.0052 (8)
N3	0.0378 (10)	0.0520 (12)	0.0409 (11)	−0.0065 (8)	0.0134 (8)	−0.0079 (9)
N4	0.0336 (9)	0.0488 (11)	0.0347 (9)	−0.0064 (8)	0.0175 (8)	−0.0098 (8)

### Geometric parameters (Å, °)

**Table:**

C1—C7	1.347 (3)	C9—C10	1.395 (3)
C1—C2	1.456 (2)	C9—C14	1.510 (3)
C1—C8	1.493 (2)	C10—C11	1.372 (3)
C2—N1	1.332 (2)	C10—H10	0.9400
C2—N4	1.335 (2)	C11—C12	1.374 (3)
C3—N1	1.348 (2)	C11—H11	0.9400
C3—N2	1.358 (2)	C12—C13	1.382 (3)
C3—C6	1.413 (3)	C12—H12	0.9400
C4—N2	1.317 (3)	C13—C15	1.507 (3)
C4—N3	1.351 (3)	C14—H14A	0.9700
C4—H4	0.9400	C14—H14B	0.9700
C5—N3	1.323 (2)	C14—H14C	0.9700
C5—C6	1.391 (3)	C15—H15A	0.9700
C5—H5	0.9400	C15—H15B	0.9700
C6—C7	1.419 (3)	C15—H15C	0.9700
C7—H7	0.9400	N4—H4A	0.8700
C8—C9	1.392 (3)	N4—H4B	0.8700
C8—C13	1.408 (3)		
			
C7—C1—C2	117.54 (17)	C9—C10—H10	119.3
C7—C1—C8	121.83 (16)	C10—C11—C12	119.9 (2)
C2—C1—C8	120.51 (16)	C10—C11—H11	120.1
N1—C2—N4	117.49 (16)	C12—C11—H11	120.1
N1—C2—C1	123.29 (17)	C11—C12—C13	121.0 (2)
N4—C2—C1	119.19 (17)	C11—C12—H12	119.5
N1—C3—N2	116.55 (16)	C13—C12—H12	119.5
N1—C3—C6	123.26 (17)	C12—C13—C8	118.86 (19)
N2—C3—C6	120.19 (17)	C12—C13—C15	120.24 (19)
N2—C4—N3	129.19 (19)	C8—C13—C15	120.89 (18)
N2—C4—H4	115.4	C9—C14—H14A	109.5
N3—C4—H4	115.4	C9—C14—H14B	109.5
N3—C5—C6	123.63 (18)	H14A—C14—H14B	109.5
N3—C5—H5	118.2	C9—C14—H14C	109.5
C6—C5—H5	118.2	H14A—C14—H14C	109.5
C5—C6—C3	116.98 (18)	H14B—C14—H14C	109.5
C5—C6—C7	125.45 (17)	C13—C15—H15A	109.5
C3—C6—C7	117.48 (17)	C13—C15—H15B	109.5
C1—C7—C6	120.61 (17)	H15A—C15—H15B	109.5
C1—C7—H7	119.7	C13—C15—H15C	109.5
C6—C7—H7	119.7	H15A—C15—H15C	109.5
C9—C8—C13	120.61 (17)	H15B—C15—H15C	109.5
C9—C8—C1	121.08 (17)	C2—N1—C3	117.76 (15)
C13—C8—C1	118.31 (17)	C4—N2—C3	115.93 (17)
C8—C9—C10	118.21 (19)	C5—N3—C4	114.02 (17)
C8—C9—C14	121.74 (17)	C2—N4—H4A	120.0
C10—C9—C14	120.05 (18)	C2—N4—H4B	120.0
C11—C10—C9	121.4 (2)	H4A—N4—H4B	120.0
C11—C10—H10	119.3		

### Hydrogen-bond geometry (Å, °)

**Table:**

*D*—H···*A*	*D*—H	H···*A*	*D*···*A*	*D*—H···*A*
N4—H4A···N1^i^	0.87	2.18	3.044 (2)	171

Symmetry codes: (i) −*x*+3/2, −*y*+1/2, −*z*+1.
